# Bone Health in Children and Adolescents with Sex Chromosome Aneuploidies: A Narrative Review

**DOI:** 10.3390/jcm15218684

**Published:** 2026-11-07

**Authors:** Roberto Paparella, Fabiola Panvino, Vittorio Maglione, Francesca Tarani, Marcello Niceta, Debora Rasio, Ida Pucarelli, Marco Fiore, Luigi Tarani

**Affiliations:** 1 Department of Maternal Infantile and Urological Sciences, Sapienza University of Rome, 00161 Rome, Italy; 2 Department of Human Neuroscience, Sapienza University of Rome, 00185 Rome, Italy; 3 Molecular Genetics and Functional Genomics, Ospedale Pediatrico Bambino Gesù, IRCCS, 00146 Rome, Italy; 4 Department of Clinical and Molecular Medicine, Sapienza University of Rome, 00189 Rome, Italy; 5 Institute of Biochemistry and Cell Biology (IBBC-CNR), c/o Department of Sensory Organs, Sapienza University of Rome, 00161 Rome, Italy

**Keywords:** sex chromosome aneuploidies, bone mineral density, Turner syndrome, Klinefelter syndrome, child, adolescent, dual-energy X-ray absorptiometry

## Abstract

**Background/Objectives**: Sex chromosome aneuploidies (SCAs)—including Turner syndrome, Klinefelter syndrome, Trisomy X (47,XXX), and 47,XYY (Jacobs) syndrome—are among the most common chromosomal conditions in humans. The available evidence on skeletal health in SCAs is heterogeneous and varies substantially across conditions. Although skeletal abnormalities and increased fracture risk are well documented in Turner syndrome, pediatric evidence in Klinefelter syndrome and Trisomy X remains limited, while dedicated skeletal data for 47,XYY are essentially absent. This narrative review summarizes current evidence on bone health in children and adolescents with the major SCAs. **Methods**: A structured literature search of PubMed/MEDLINE, Embase, and the Cochrane Library was conducted, supplemented by backward and forward citation tracking. Pediatric studies were prioritized, while adult and mechanistic studies were included when necessary to address evidence gaps. Owing to the narrative design, no formal risk-of-bias assessment or quantitative synthesis was performed. **Results**: Pediatric evidence is most extensive for Turner syndrome, in which fracture risk and bone mineral status are influenced by ovarian insufficiency, growth, body size, comorbidities, and treatment exposure. Direct pediatric skeletal evidence for Klinefelter syndrome and Trisomy X remains limited and heterogeneous, deriving from small observational studies and, for Klinefelter syndrome, a randomized interventional trial. Dedicated data for 47,XYY syndrome are essentially absent. Interpretation of dual-energy X-ray absorptiometry requires particular attention to short stature, tall stature, and pubertal delay. **Conclusions**: Clinical assessment should be individualized, with attention to endocrine and nutritional factors, appropriate interpretation of bone imaging, and promotion of bone-healthy lifestyle measures. Further prospective pediatric studies are needed to clarify fracture risk, monitoring strategies, and treatment effects across SCAs.

## 1. Introduction

Peak bone mass (PBM)—the maximum bone quantity attained at skeletal maturity—is a principal determinant of lifelong fracture risk, and up to 40% of its variance is attributable to modifiable lifestyle and hormonal factors acting during childhood and adolescence [1,2]. Bone mass roughly doubles across puberty, with the late-childhood and peripubertal years representing the period of most rapid accrual [3]. Conditions that disrupt pubertal timing, sex-steroid exposure, nutrition, or physical activity during this window can compromise PBM and accelerate the later onset of osteoporosis. The importance of optimizing bone mass acquisition during childhood and adolescence extends beyond the attainment of PBM, as skeletal health throughout adulthood contributes to the prevention of fragility fractures. Vertebral fractures, in particular, represent a substantial global public-health burden, with approximately 5.37 million prevalent cases estimated worldwide in 2021 [4].

Sex chromosome aneuploidies (SCAs) intersect with several of these determinants simultaneously. Hypogonadism (Turner and Klinefelter syndromes), gonadotropin excess, hypovitaminosis D, altered body composition, short or tall stature, and possible intrinsic effects of aberrant sex-chromosome gene dosage all plausibly contribute to a skeletal disadvantage that begins in childhood [5,6]. Because two of the four common SCAs (Klinefelter and Trisomy X) remain undiagnosed in the majority of affected individuals, and because the expansion of cell-free DNA prenatal screening is rapidly increasing ascertainment of previously silent cases, pediatricians and pediatric endocrinologists are encountering these patients earlier and in greater numbers [7,8]. This review summarizes what is known—and the substantial gaps in what is known—about bone health in children and adolescents with SCAs.

## 2. Methods

This article is a narrative (non-systematic) review. Searches of PubMed/MEDLINE, Embase, and the Cochrane Library were conducted from database inception through August 2026, using combinations of controlled vocabulary (MeSH/Emtree) and free-text terms for the SCAs and for skeletal outcomes. Population terms included “Turner syndrome,” “45,X,” “Klinefelter syndrome,” “47,XXY,” “Trisomy X,” “triple X syndrome,” “47,XXX,” “47,XYY,” “Jacobs syndrome,” “sex chromosome aneuploidy,” and “sex chromosome trisomy.” These were combined (Boolean AND) with outcome terms including “bone mineral density,” “bone density,” “osteoporosis,” “osteopenia,” “fracture,” “bone fragility,” “peak bone mass,” “DXA/dual-energy X-ray absorptiometry,” “quantitative computed tomography,” “peripheral QCT,” “trabecular bone score,” “bone turnover markers,” “vitamin D,” “estrogen replacement,” and “growth hormone.” No language restriction was applied, although the final evidence base was predominantly English-language. Reference lists of included articles, relevant clinical practice guidelines, and consensus statements were hand-searched to identify additional sources (backward citation tracking), and forward citation searches were performed for pivotal studies. Priority was given to studies enrolling children and adolescents (age < 18 years), but seminal adult and mechanistic studies were included where pediatric data were absent or necessary to explain pathophysiology and later-life consequences. Eligible sources comprised original research (cross-sectional, cohort, and interventional studies), systematic reviews and meta-analyses, clinical practice guidelines and international consensus statements, and mechanistic/basic-science reports. Case reports were included only when they provided otherwise-unavailable information on rare karyotypes; conference abstracts were considered only when they contributed data not available in full-text form.

Given the narrative design, no formal risk-of-bias scoring, quantitative synthesis, or PRISMA-based selection protocol was applied. Study selection was performed by two review authors (R.P. and F.P.), who assessed the relevance of retrieved publications based on their titles, abstracts, and full-text content. When multiple studies addressed similar outcomes, priority was given to evidence with greater direct relevance to pediatric populations, larger or longitudinal cohorts, and more robust skeletal assessment methods. Study design, sample size, age and pubertal status, treatment exposure, recruitment setting, and outcome definition were considered when interpreting the findings. When results were conflicting, these differences were taken into account rather than assuming that the findings were directly comparable. Adult studies and mechanistic evidence were used primarily to provide context where pediatric evidence was limited. This narrative approach does not aim to provide an exhaustive systematic assessment of all available studies and may be subject to selection and citation bias.

## 3. Epidemiology and Skeletal Overview

Pooled newborn cytogenetic surveys estimate the prevalence of Turner syndrome at approximately 50 per 100,000 females (~1 in 2000), Klinefelter syndrome at approximately 150 per 100,000 males (~1 in 660), Trisomy X at approximately 85 per 100,000 females (~1 in 1000), and 47,XYY at approximately 98 per 100,000 males (~1 in 1000) [9]. Diagnosis rates diverge sharply from these biological frequencies: Turner syndrome has a substantially higher diagnostic rate than the sex chromosome trisomies, although mosaic and less clinically apparent cases may remain undiagnosed, while only about 38% of Klinefelter, 13% of Trisomy X, and 18% of 47,XYY cases are ever identified, meaning the published skeletal literature is drawn from a clinically ascertained—and likely more severely affected—minority [8]. Large biobank analyses confirm this profound under diagnosis for the supernumerary trisomies [8,10,11].

The skeletal phenotypes differ by karyotype and are summarized in Table 1. Turner syndrome combines short stature (*SHOX* haploinsufficiency), ovarian dysgenesis with hypergonadotropic hypogonadism, and a well-documented increase in fracture risk. Klinefelter syndrome combines tall stature, progressive testicular failure, and adult osteopenia/osteoporosis. Trisomy X and 47,XYY are characterized by tall stature; the former has emerging evidence of impaired childhood bone mineral status, while the latter has almost no dedicated skeletal data despite registry evidence of increased musculoskeletal and traumatic morbidity [9].

## 4. Turner Syndrome

### 4.1. Fracture Risk and Bone Mineral Density

Turner syndrome, including 45,X and related mosaic or structural X-chromosome abnormalities, is the SCA for which skeletal fragility and fracture risk have been most extensively investigated. However, the available estimates derive from populations and study designs that should be distinguished from direct assessments of pediatric fracture risk. Epidemiologic data indicate a lifetime fracture risk approximately 1.4- to 2.2-fold higher than in the general population, with a bimodal age distribution—a childhood peak (particularly forearm and wrist fractures) and a later post-menopausal peak related to osteoporosis [12]. These estimates describe the broader lifespan and should not be interpreted as quantitative estimates of fracture risk specifically during childhood. Adult-focused reviews have also reported that fracture risk appears to increase with age, with a particularly marked burden in adulthood [16].

Pediatric evidence is more heterogeneous. In a U.S. national patient survey, overall fracture prevalence in participants younger than 25 years did not differ from peers, but adolescents with Turner syndrome reported significantly more forearm fractures, and women aged 45 years or older had a greater cumulative fracture burden [17]. The survey design and broad age category limit the interpretation of these findings as estimates of fracture risk specifically in children.

Importantly, contemporary cohorts treated with growth hormone (GH) and timely estrogen may have a substantially attenuated childhood fracture risk. A 6-year prospective peripheral quantitative computed tomography (pQCT) study found that although trabecular volumetric bone mineral density (BMD) Z-scores declined with age, a compensatory increase in total bone cross-sectional area maintained normal bone strength, and the observed fracture rate was not substantially higher than in controls [18]. These findings suggest that bone density, bone geometry, estimated strength, and clinical fracture incidence do not necessarily follow parallel trajectories. However, the absence of a substantial fracture excess in this cohort does not establish that contemporary treatment normalizes fracture risk. Differences in treatment exposure, follow-up duration, and statistical power should be considered when comparing contemporary pediatric cohorts with historical or lifetime epidemiological estimates [19].

### 4.2. Pathophysiology

Bone fragility in Turner syndrome is multifactorial. Estrogen deficiency from ovarian insufficiency is the dominant contributor, impairing both peripubertal accrual and later maintenance of bone mass; girls with spontaneous menarche have higher BMD than those with absent menarche. However, a cortical deficit associated with a low-turnover state is detectable in childhood before the expected age of puberty, implying additional mechanisms [16].

Gonadotropin excess is an increasingly recognized independent contributor. Peripheral blood mononuclear cells cultured from girls with Turner syndrome and high follicle-stimulating hormone (FSH) levels show enhanced spontaneous osteoclastogenesis, driven by monocytes expressing elevated c-fms, RANK, and TNF-α, and by T-cells producing high RANKL [20]. These findings are consistent with experimental evidence suggesting that FSH may exert direct, estrogen-independent effects on osteoclast formation and survival via an FSH receptor splice variant on bone cells [21,22]. However, these experimental findings provide biological plausibility for a potential role of FSH in bone metabolism, but do not establish its independent contribution to bone loss or fracture risk in children with Turner syndrome. The clinical relevance and relative contribution of this pathway to skeletal fragility remain uncertain. An intrinsic skeletal effect of X-chromosome haploinsufficiency is also postulated, though a 2016 study found no effect of specific karyotype on bone mineral status [23]. Comorbidities—vitamin D deficiency, celiac disease, inflammatory bowel disease, thyroid disease, and hearing loss with attendant fall risk—further compound fracture risk [19,24].

### 4.3. Hormonal Management and Bone

Estrogen replacement is a key component of endocrine management in Turner syndrome and is important for physiological pubertal development and bone accrual. Current international guidance recommends individualized pubertal induction with low-dose estradiol, generally beginning around the usual age of pubertal onset when ovarian insufficiency is established and spontaneous pubertal development is absent, followed by gradual dose escalation over approximately 2–3 years [25]; delaying replacement is deleterious to both bone and uterine health [26]. A meta-analysis confirmed that estrogen replacement significantly increases lumbar spine BMD [27]. Later age at initiation of estrogen-progestin therapy is independently associated with lower young adult BMD [28]. Transdermal estradiol is generally preferred over oral or ethinyl estradiol; conjugated estrogens are specifically discouraged [26]. Emerging interest centers on whether even earlier, ultra-low-dose transdermal estradiol—guided by rising FSH as a marker of estrogen deficiency—could better approximate endogenous secretion and improve bone outcomes, though this remains unproven [29]. Notably, the rate of dose escalation has not been shown to significantly alter BMD accrual [30].

GH, standard of care for short stature in Turner syndrome, has a more modest and less consistent skeletal effect. GH increases lean body mass and reduces adiposity but, after adjustment for bone size and bone age, has little independent effect on cortical or trabecular BMD [31]; a meta-analysis found no significant BMD benefit from GH alone [27]. Nonetheless, by increasing bone size and cross-sectional area, GH may improve bone strength and geometry, and adequate, timely GH plus appropriately timed pubertal induction appears sufficient for age-appropriate bone accrual in many patients [19,32].

## 5. Klinefelter Syndrome (47,XXY)

### 5.1. Bone Mineral Status from Childhood Through Adulthood

Klinefelter syndrome is the most frequent SCA in males, described by a karyotype with one or more extra X Chromosome (most frequently 47,XXY) [33]. In adults with Klinefelter syndrome, densitometric osteoporosis affects an estimated 6–15% and osteopenia 25–48%, and vertebral fractures are found in up to 15–20% of patients—often independent of testosterone levels and areal BMD. Femoral fractures are an important cause of morbidity and mortality [13]. These estimates describe adult populations and should not be extrapolated directly to children and adolescents. Adult studies nevertheless provide important context for understanding the potential long-term skeletal consequences of the syndrome.

Alterations in bone-related measurements and body composition have also been reported during childhood and adolescence [34], although the available evidence remains limited and heterogeneous. A cross-sectional study of children and adolescents with Klinefelter syndrome, using phalangeal quantitative ultrasound (amplitude-dependent speed of sound and bone transmission time), found significantly reduced Z-scores compared with healthy controls, with more than 17% of patients having Z-scores at or below −2.0. Lower 25-hydroxyvitamin D concentrations, higher parathyroid hormone (PTH) levels, and reduced bone formation markers were also reported before puberty [35]. These findings suggest that differences in skeletal-related measurements and mineral metabolism may already be detectable during childhood. However, phalangeal quantitative ultrasound parameters are not equivalent to dual-energy X-ray absorptiometry (DXA)-derived areal BMD or volumetric BMD, and this observational study cannot establish increased fracture risk or determine whether the observed differences are independent of nutritional, endocrine, developmental, or body-composition factors.

Other studies have provided additional, but not entirely consistent, evidence. In a study of 24 boys with Klinefelter syndrome (median age 11.0 years, range 4.3–18.6), Aksglaede et al. reported normal lumbar spine BMD and total-body bone mineral content (BMC), despite increased fat mass and an unfavorable muscle-to-fat ratio [36]. In a randomized, placebo-controlled trial involving boys aged 4–12 years, Vogiatzi et al. found a significantly greater improvement in the Bone Health Index, assessed by digital X-ray radiogrammetry, after two years of oxandrolone treatment compared with placebo [37]. Although this provides interventional evidence of an effect on a radiogrammetric bone parameter, it does not establish oxandrolone as a routine treatment for skeletal abnormalities in Klinefelter syndrome. More recently, Krabbe et al. reported normal total-body BMC but lower BMC after adjustment for bone area in a cohort of 62 patients aged 5.9–20.6 years [38]. Similarly, Spiekermann et al. reported lower mean Bone Health Index and lumbar spine BMD Z-scores in a pilot cohort of 20 individuals aged 9.3–25.4 years. The latter two cohorts included young adults and should therefore not be interpreted as exclusively pediatric evidence. Overall, these studies suggest that alterations in skeletal measurements and body composition may be detectable from childhood, but findings vary according to the assessment method and study population. Small sample sizes and the inclusion of mixed-age populations limit the interpretation and generalizability of the available evidence.

Additional evidence comes from young, testosterone-naïve adults studied using high-resolution pQCT (HR-pQCT), which demonstrated marked cortical impairment at the radius and combined cortical and trabecular deficits at the tibia [39]. These findings provide information on adult bone microarchitecture but do not constitute direct evidence of the same abnormalities in pediatric populations. Finally, endocrine and metabolic abnormalities have also been documented in prepubertal boys with 47,XXY [40]; however, such findings should be distinguished from direct measurements of bone density, bone microarchitecture, or fracture incidence.

### 5.2. Pathophysiology

Historically attributed solely to hypogonadism, low bone mass in Klinefelter syndrome is now understood as multifactorial. Low BMD and fragility fractures occur even in patients with normal testosterone, and testosterone replacement does not fully normalize bone parameters [13]. Hypovitaminosis D may represent one of several potential contributors to skeletal abnormalities. Lower 25-hydroxyvitamin D levels have been reported in comparison with controls, including in prepubertal patients [35,41]. However, findings are not uniform: in a recent pilot study, bone turnover markers were largely within age-appropriate ranges, and PTH levels were generally normal, despite vitamin D deficiency in some participants [42]. These findings highlight the heterogeneity of the available evidence and do not establish vitamin D deficiency as a predominant determinant of skeletal abnormalities. Reduced muscle strength and lean mass, elevated FSH, bone-turnover markers, and possibly low insulin-like peptide 3 (a Leydig-cell product and negative regulator of osteocyte sclerostin) might represent additional contributing factors; in one adult cohort, muscle strength and bone markers—but not testosterone itself—independently predicted BMD [43,44].

### 5.3. Management

Testosterone replacement therapy (TRT) is indicated for clinically significant hypogonadism according to established endocrine criteria and may improve body composition and lumbar-spine BMD [45]; treatment decisions should be guided by the clinical and biochemical assessment of gonadal function, with fertility preservation considered beforehand [13]. The European Academy of Andrology guidelines recommend regular assessment of pubertal development, testosterone and gonadotropin levels, and signs and symptoms of hypogonadism in boys with Klinefelter syndrome, starting before the expected onset of puberty. In adolescents, testosterone supplementation is recommended in cases of delayed puberty and/or symptoms of hypogonadism associated with low-normal testosterone and elevated luteinizing hormone levels, after fertility issues have been addressed. Conversely, testosterone therapy is not recommended in adolescents with compensated hypergonadotropic hypogonadism [33]. Although these recommendations provide syndrome-specific clinical guidance, uncertainties remain regarding the optimal timing and regimen of treatment and its long-term skeletal effects. Fertility status and preservation options should be discussed with patients and, when appropriate, their families before treatment initiation. Nonspecific findings such as low muscle tone or gynecomastia alone do not constitute indications for TRT.

The timing and severity of testicular dysfunction are important determinants when considering the potential contribution of hypogonadism to skeletal health in 47,XXY [46]. Vitamin D deficiency should be identified and corrected according to age-appropriate pediatric recommendations, alongside appropriate lifestyle measures [41,44]. However, correction of a documented nutritional deficiency should be distinguished from testosterone treatment for clinically indicated hypogonadism, and the available evidence does not allow their relative contribution to skeletal outcomes in children and adolescents with Klinefelter syndrome to be ranked.

## 6. Trisomy X (47,XXX)

Trisomy X is the most common female chromosomal abnormality (~1 in 1000 female births) but is diagnosed in only about 10% of cases; the phenotype includes tall stature, epicanthal folds, hypotonia, clinodactyly, scoliosis, learning and language difficulties, and premature ovarian insufficiency in a subset [47,48].

Direct evidence on skeletal health in Trisomy X is very limited and derives primarily from a single cross-sectional study of 19 girls with non-mosaic Trisomy X size- and age-matched controls. The study reported significantly lower phalangeal ultrasound Z-scores in both prepubertal and pubertal subgroups, accompanied by lower ionized calcium and 25-hydroxyvitamin D and higher phosphate and PTH levels [14]. These findings indicate reduced phalangeal quantitative ultrasound measurements and concomitant biochemical differences; however, these measurements are not equivalent to DXA-derived bone mineral density and do not establish osteoporosis or increased fracture risk. The clinical significance of these observations and their potential relationship with X-chromosome dosage remain uncertain. Nevertheless, the small sample size and cross-sectional design do not establish a primary chromosome-dosage-related skeletal deficit or demonstrate that the observed differences are independent of nutritional status, endocrine factors, pubertal development, physical activity, muscle function, or body composition. Premature ovarian insufficiency, which can develop in a subset of individuals with Trisomy X, is an additional consideration for longitudinal clinical assessment [48]. At present, the available evidence is insufficient to establish a Trisomy X-specific bone-screening schedule; assessment should instead be guided by clinical findings and individual risk factors.

## 7. 47,XYY Syndrome and Higher-Grade Polysomies

There is essentially no dedicated pediatric skeletal literature for 47,XYY. The phenotype includes tall stature, macrocephaly, hypotonia, and possible macro-orchidism, with generally preserved gonadal function distinguishing it from Klinefelter syndrome [15]. A Danish registry study of males with 47,XYY found significantly increased hospital encounters for musculoskeletal and connective-tissue disease and for trauma, alongside broader multisystem morbidity [9]. These registry observations identify a possible musculoskeletal burden but cannot distinguish primary skeletal fragility from differences in trauma exposure, motor function, comorbidity, or healthcare ascertainment.

Mechanistically, induced pluripotent stem cell models of 47,XYY reveal a transcriptomic signature shared with 47,XXY, mediated in part by feedback between X- and Y-chromosome gene homologs (e.g., the *UTY*–*KDM6A* pair) [49]. These findings suggest potentially shared molecular pathways between male SCAs; however, their relevance to skeletal development and the existence of a shared bone phenotype remain unestablished. Higher-grade polysomies (48,XXYY; 48,XXXY; 49,XXXXY) are individually rare and lack systematic skeletal data. For all of these, bone health recommendations are necessarily extrapolated from Klinefelter syndrome and general pediatric bone principles.

## 8. Assessment of Bone Health in the Growing SCA Skeleton

Accurate skeletal assessment is uniquely challenging in SCAs because the two most affected conditions distort body size in opposite directions (short stature in Turner, tall stature in Klinefelter and the trisomies) and because pubertal delay is common. Table 2 compares the principal modalities.

DXA remains the most widely used and available technique and is the pediatric method of choice, with lumbar spine and total-body-less-head as preferred sites and results expressed as age- and sex-specific Z-scores rather than T-scores in children and adolescents [51,52]. Its central limitation is that areal BMD (g/cm^2^) is a two-dimensional projection that does not account for bone depth: for two bones of identical true volumetric density, the smaller one yields a lower areal BMD. Consequently, DXA systematically underestimates BMD in short-statured or pubertally delayed children—precisely the Turner syndrome population—and can overestimate it in tall children [50,54].

The International Society for Clinical Densitometry [50] recommends adjusting DXA measurements for body size in children with short stature or growth delay. The recommended approach is site-specific: lumbar spine measurements may be adjusted using bone mineral apparent density or height Z-score, whereas total-body-less-head measurements should be adjusted using height Z-score; freely available height-adjusted pediatric reference calculators exist [50,51]. No single method is universally endorsed, and a caveat of height-age adjustment is that short-for-age children are then compared to younger children at an earlier maturational stage. These approaches should not be considered interchangeable, and the choice should follow pediatric densitometry recommendations. Although volumetric techniques can help distinguish bone-size effects from differences in volumetric density, their availability and pediatric reference data remain limited.

pQCT and HR-pQCT provide volumetric density measurements and allow separate assessment of cortical and trabecular compartments and bone geometry, thereby reducing some of the limitations associated with areal BMD. However, these techniques do not eliminate all effects of growth, skeletal maturation, and bone geometry, which remain relevant to the interpretation of pediatric measurements. They have been pivotal in showing that apparent DXA “osteopenia” in Turner syndrome may be offset by increased bone cross-sectional area yielding normal strength [18,19]. The ACR–SPR–SSR practice parameter explicitly lists Turner syndrome among endocrine indications for quantitative computed tomography (QCT) and notes QCT’s particular utility in children whose short stature or spinal deformity degrades DXA accuracy [53]. Trabecular bone score and HR-pQCT add microarchitectural detail relevant to fracture risk beyond areal BMD, which itself explains only 60–70% of bone strength [12,44]. These modalities remain largely research tools with limited normative pediatric data.

A critical interpretive point applies across all SCAs: in children, the terms “osteopenia” and “osteoporosis” should not be assigned on densitometry alone. A BMD Z-score ≤ −2.0 is classified as “low bone mineral density for age.” Importantly, a BMD Z-score above −2.0 does not exclude skeletal fragility or increased fracture risk, and densitometric findings should always be interpreted in the context of clinical history and fracture history. According to pediatric criteria, osteoporosis may be diagnosed in either of two circumstances: (1) the presence of one or more vertebral compression fractures, in the absence of local disease or high-energy trauma, regardless of BMD; or (2) a clinically significant fracture history (≥2 long-bone fractures by age 10 years or ≥3 by age 19 years) together with a BMD Z-score ≤ −2.0 [51].

## 9. Management Principles and Monitoring

### 9.1. Foundational (All SCAs)

Optimization of modifiable determinants of PBM applies to every child with an SCA, given that lifestyle factors influence 20–40% of adult PBM [1]. Age-appropriate calcium intake (1300 mg/day for ages 9–18) should be met preferentially through diet; routine calcium supplementation is not advised for otherwise healthy children [51,55]. Vitamin D deficiency should be identified and treated according to age-appropriate pediatric guidance. Although adult studies in Klinefelter syndrome suggest that correction of vitamin D deficiency may improve BMD, the optimal vitamin D formulation, target concentration, and supplementation strategy in children and adolescents with SCAs remain uncertain [41,51,56]. Regular weight-bearing and high-impact activities (e.g., jumping programs) produce measurable gains in bone mass, with the strongest evidence during the peripubertal years [1,57].

### 9.2. Condition-Specific Hormonal Therapy

Hormonal treatment should follow established syndrome-specific endocrine indications. In Turner syndrome, this includes timely pubertal induction with transdermal estradiol when ovarian insufficiency is established and spontaneous pubertal development is absent [26]. In Klinefelter syndrome, testosterone replacement should be considered in adolescents/men with clinically evident hypogonadism [13]. Although sex-steroid replacement is important for normal bone accrual, its specific effect on fracture prevention in pediatric SCAs has not been established. GH is indicated for short stature in Turner syndrome and may contribute to bone size and strength even if its direct BMD effect is modest [31].

### 9.3. Pharmacologic Antiresorptive Therapy

Pharmacologic options for pediatric bone fragility are limited and are generally considered for children with clinically significant fractures despite optimization of the measures described above. Bisphosphonates represent the mainstay of pharmacologic treatment for pediatric osteoporosis and bone fragility, whereas denosumab has been used only in selected refractory pediatric conditions, with limited evidence and important safety considerations [58,59]. Notably, the growing pediatric skeleton has a unique capacity for spontaneous recovery from bone fragility, including vertebral reshaping, which should be considered when determining the need and timing of pharmacologic treatment [52]. There are no pediatric trials specifically evaluating antiresorptive therapy in SCAs, and the role of such treatment in preventing fractures in children and adolescents with Turner syndrome remains undefined [19]. Most antiresorptive therapies used for osteoporosis are approved primarily for adult populations, further limiting their evidence base and routine use in pediatric patients.

### 9.4. Monitoring

For Turner syndrome, the 2024 international clinical practice guidelines recommend obtaining a DXA scan after completion of growth but before 21 years of age, followed by assessment every 5–10 years in adulthood. More frequent monitoring is recommended in individuals with additional risk factors, including fractures, inadequate hormone replacement, celiac disease, or other relevant comorbidities [25]. In Klinefelter syndrome, the European Academy of Andrology guidelines suggest measuring vitamin D levels and ensuring adequate vitamin D and calcium intake or supplementation in prepubertal boys. Assessment of bone mineral status by DXA every two years is suggested in children with vitamin D deficiency; however, this recommendation is based on limited evidence [33]. For Trisomy X, the available pediatric evidence does not establish a specific bone-screening schedule. DXA and biochemical evaluation should therefore be guided by clinical findings and individual risk factors [14].

## 10. Knowledge Gaps and Future Directions

The interpretation of the available evidence is further limited by potential referral and ascertainment biases. Clinically recruited cohorts may overrepresent individuals with more pronounced phenotypes or skeletal concerns, whereas prenatally identified individuals may include a broader spectrum of clinical manifestations. Moreover, treatment-related confounding cannot be excluded, as individuals receiving hormonal or other interventions may differ at baseline from untreated patients in terms of disease severity, endocrine status, and associated risk factors. These limitations, together with small sample sizes and the inclusion of mixed pediatric and adult populations in some studies, restrict the generalizability of the available findings and preclude firm conclusions regarding the independent contribution of individual factors to skeletal outcomes. Moreover, several priorities emerge. First, the pediatric evidence base is heavily skewed toward Turner syndrome; robust, prospective, size-adjusted studies in childhood Klinefelter and Trisomy X—using volumetric BMD/HR-pQCT rather than ultrasound alone—are needed, and dedicated skeletal data for 47,XYY and higher-grade polysomies are almost entirely lacking. Second, the optimal timing and formulation of pubertal induction for maximizing bone outcomes in Turner syndrome (including whether earlier ultra-low-dose transdermal estradiol confers benefit) remains unresolved [29]. Third, the relative contributions of hypovitaminosis D, gonadotropin excess, muscle deficit, and intrinsic sex chromosome gene dosage require mechanistic clarification, particularly given evidence that FSH directly drives osteoclastogenesis [22]. Fourth, whether contemporary treatment normalizes fracture risk—as some Turner data now suggest—needs confirmation in prospectively followed, prenatally ascertained cohorts, which the expansion of cell-free DNA screening will make feasible [7]. Finally, the role, if any, of antiresorptive therapy in SCA-related pediatric bone fragility is undefined.

## 11. Conclusions

Skeletal abnormalities in children with SCAs are likely multifactorial, involving varying contributions from gonadal dysfunction, nutritional and metabolic factors, altered body composition, and comorbidities. Intrinsic effects of sex-chromosome gene dosage represent a plausible additional mechanism, although their independent contribution to pediatric skeletal impairment remains insufficiently established. Turner syndrome carries the best-characterized evidence of skeletal involvement and fracture risk. Limited and heterogeneous pediatric evidence suggests alterations in skeletal measurements and body composition in Klinefelter syndrome, based on observational and interventional studies using different assessment methods. However, these findings do not establish the extent of skeletal fragility or pediatric fracture risk. Evidence in Trisomy X remains limited to small observational studies. In contrast, adequate direct evidence of altered bone mineral status or increased skeletal fragility in children with 47,XYY is currently lacking; musculoskeletal morbidity data and experimental findings should not be interpreted as establishing a pediatric skeletal phenotype. Assessment demands attention to the size-confounding limitations of DXA and appropriate use of height adjustment and volumetric techniques. Clinical management should be individualized, focusing on the timely treatment of documented endocrine abnormalities, correction of nutritional deficiencies, adequate calcium intake, weight-bearing physical activity, and skeletal monitoring when clinically indicated. Larger prospective pediatric studies are needed to clarify skeletal outcomes and inform evidence-based surveillance and management strategies across SCAs.

## Figures and Tables

**Table 1:** Epidemiology and skeletal phenotype across the common SCAs [8,9,12,13,14,15].

Feature	Turner (45,X)	Klinefelter (47,XXY)	Trisomy X (47,XXX)	47,XYY
Approximate birth prevalence	~1 in 2000 females	~1 in 500–660 males	~1 in 1000 females	~1 in 1000 males
Proportion ever diagnosed	Most; mosaic and less clinically apparent forms may remain undiagnosed	~38%	~10–13%	~18%
Stature	Short (*SHOX*)	Tall	Tall	Tall
Gonadal status	Hypergonadotropic hypogonadism	Progressive primary hypogonadism	Usually preserved; premature ovarian insufficiency in subset	Usually preserved
Fracture and bone-measurement evidence	Increased fracture risk (~1.4–2.2-fold; predominantly lifetime estimates)	Mostly adult vertebral fracture data (15–20%); limited and heterogeneous pediatric evidence from ultrasound, DXA-based studies, and radiogrammetry; no established pediatric fracture-risk data	Limited pediatric evidence; one cohort reporting altered ultrasound-derived skeletal parameters	No adequate direct skeletal evidence
Earliest reported skeletal findings	Prepubertal cortical bone abnormalities in pediatric studies	Prepubertal biochemical and cortical bone findings; evidence from small pediatric cohorts	Prepubertal differences in phalangeal quantitative ultrasound measurements in one small pediatric cohort	Unknown; no dedicated pediatric skeletal studies
Potential contributors and proposed mechanisms	Estrogen deficiency; possible contribution of FSH and X-chromosome haploinsufficiency	Hypovitaminosis D, hypogonadism, altered body composition; possible contribution of gonadotropin excess	Reported vitamin D and PTH abnormalities; possible chromosome-dosage effects	Insufficient evidence; shared mechanisms with 47,XXY remain speculative

Abbreviations: DXA, dual-energy X-ray absorptiometry; FSH, follicle-stimulating hormone; PTH, parathormone.

**Table 2:** Bone assessment modalities in children with SCAs [18,50,51,52,53].

Modality	Strengths	Limitations in SCAs	Role
DXA (areal BMD)	Widely available, low dose, pediatric norms, reproducible	Size confounded—underestimates in short stature (Turner), overestimates in tall stature; requires height/height-age or volumetric BMD adjustment	First-line; use Z-scores, never T-scores in children
Height-adjusted DXA/bone mineral apparent density	Helps account for the influence of body size on areal BMD	The appropriate method depends on the skeletal site; no single approach applies universally	Size adjustment when indicated by short stature or growth delay
Volumetric techniques (pQCT/HR-pQCT)	True volumetric BMD; separates cortical vs. trabecular; geometry and strength	Limited pediatric norms; less available; research-oriented; measurements remain influenced by growth, maturation, and skeletal geometry	Clarifies whether low areal BMD reflects true deficit vs. size
Trabecular bone score	Adds microarchitecture beyond areal BMD	Limited pediatric validation	Adjunct to DXA for fracture-risk refinement
QCT	Volumetric; useful with spinal deformity or poor DXA cooperation	Higher radiation; Turner listed as an indication	Selected cases

Abbreviations: BMD, bone mineral density; DXA, dual-energy X-ray absorptiometry; QCT, quantitative computed tomography; pQCT, peripheral QCT; HR-pQCT, high-resolution pQCT; SCAs, sex chromosome aneuploidies.

## Data Availability

No new data were created or analyzed in this study. Data sharing is not applicable to this article.

## Abbreviations

The following abbreviations are used in this manuscript:

BMC	Bone mineral content
BMD	Bone mineral density
DXA	Dual-energy X-ray absorptiometry
FSH	Follicle-stimulating hormone
GH	Growth hormone
HR-pQCT	High-resolution peripheral quantitative computed tomography
PBM	Peak bone mass
pQCT	Peripheral quantitative computed tomography
PTH	Parathyroid hormone
QCT	Quantitative computed tomography
SCAs	Sex chromosome aneuploidies
TRT	Testosterone replacement therapy

## References

[1] Weaver C.M., Gordon C.M., Janz K.F., Kalkwarf H.J., Lappe J.M., Lewis R., O’Karma M., Wallace T.C., Zemel B.S. (2016). The National Osteoporosis Foundation’s Position Statement on Peak Bone Mass Development and Lifestyle Factors: A Systematic Review and Implementation Recommendations. Osteoporos. Int. J. Establ. Result Coop. Eur. Found. Osteoporos. Natl. Osteoporos. Found. USA.

[2] Chevalley T., Rizzoli R. (2022). Acquisition of Peak Bone Mass. Best Pract. Res. Clin. Endocrinol. Metab..

[3] Stokes G., Herath M., Samad N., Trinh A., Milat F. (2025). Bone Health—Across a Woman’s Lifespan. Clin. Endocrinol..

[4] Bae Y., Kim M., Jeong W., Jang S., Lee S.W. (2025). Epidemiology and Future Burden of Vertebral Fractures: Insights from the Global Burden of Disease 1990–2021. Healthcare.

[5] Paparella R., Panvino F., Tarani F., D’Agostino B., Leonardi L., Ferraguti G., Venditti S., Colloridi F., Pucarelli I., Tarani L. (2025). An Overview of Oxidative Stress in Sex Chromosome Aneuploidies in Pediatric Populations. Antioxidants.

[6] Spaziani M., Carlomagno F., Tarantino C., Angelini F., Paparella R., Tarani L., Putotto C., Badagliacca R., Pozza C., Isidori A.M. (2024). From Klinefelter Syndrome to High Grade Aneuploidies: Expanding the Gene-Dosage Effect of Supernumerary X Chromosomes. J. Clin. Endocrinol. Metab..

[7] Tartaglia N., Davis S., Howell S., Bothwell S., Nocon K., Kowal K., Ikomi C., Keene A., Reynolds V., Berglund A. (2025). Medical Findings in Infants Prenatally Identified With Sex Chromosome Trisomy in Year 1 of Life. Pediatrics.

[8] Berglund A., Viuff M.H., Skakkebæk A., Chang S., Stochholm K., Gravholt C.H. (2019). Changes in the Cohort Composition of Turner Syndrome and Severe Non-Diagnosis of Klinefelter, 47,XXX and 47,XYY Syndrome: A Nationwide Cohort Study. Orphanet J. Rare Dis..

[9] Berglund A., Stochholm K., Gravholt C.H. (2020). The Epidemiology of Sex Chromosome Abnormalities. Am. J. Med. Genet. C Semin. Med. Genet..

[10] Zhao Y., Gardner E.J., Tuke M.A., Zhang H., Pietzner M., Koprulu M., Jia R.Y., Ruth K.S., Wood A.R., Beaumont R.N. (2022). Detection and Characterization of Male Sex Chromosome Abnormalities in the UK Biobank Study. Genet. Med..

[11] Tuke M.A., Ruth K.S., Wood A.R., Beaumont R.N., Tyrrell J., Jones S.E., Yaghootkar H., Turner C.L.S., Donohoe M.E., Brooke A.M. (2019). Mosaic Turner Syndrome Shows Reduced Penetrance in an Adult Population Study. Genet. Med..

[12] Ikegawa K., Hasegawa Y. (2022). Fracture Risk, Underlying Pathophysiology, and Bone Quality Assessment in Patients with Turner Syndrome. Front. Endocrinol..

[13] Blackburn J., Ramakrishnan A., Graham C., Bambang K., Sriranglingam U., Senniappan S. (2025). Klinefelter Syndrome: A Review. Clin. Endocrinol..

[14] Stagi S., Di Tommaso M., Scalini P., Sandini E., Masoni F., Chiarelli F., Verrotti A., Giglio S., Romano S., De Martino M. (2017). Cross-sectional Study Shows That Impaired Bone Mineral Status and Metabolism Are Found in Nonmosaic Triple X Syndrome. Acta Paediatr..

[15] Hong D.S., Reiss A.L. (2014). Cognitive and Neurological Aspects of Sex Chromosome Aneuploidies. Lancet Neurol..

[16] Gravholt C.H., Viuff M.H., Brun S., Stochholm K., Andersen N.H. (2019). Turner Syndrome: Mechanisms and Management. Nat. Rev. Endocrinol..

[17] Wasserman H., Backeljauw P.F., Khoury J.C., Kalkwarf H.J., Gordon C.M. (2018). Bone Fragility in Turner Syndrome: Fracture Prevalence and Risk Factors Determined by a National Patient Survey. Clin. Endocrinol..

[18] Soucek O., Schönau E., Lebl J., Willnecker J., Hlavka Z., Sumnik Z. (2018). A 6-Year Follow-Up of Fracture Incidence and Volumetric Bone Mineral Density Development in Girls with Turner Syndrome. J. Clin. Endocrinol. Metab..

[19] Lin A.E., Prakash S.K., Andersen N.H., Viuff M.H., Levitsky L.L., Rivera-Davila M., Crenshaw M.L., Hansen L., Colvin M.K., Hayes F.J. (2019). Recognition and Management of Adults with Turner Syndrome: From the Transition of Adolescence through the Senior Years. Am. J. Med. Genet. A..

[20] Faienza M.F., Brunetti G., Ventura A., Piacente L., Messina M.F., De Luca F., Ciccarelli M., Oranger A., Mori G., Natale M.P. (2015). Mechanisms of Enhanced Osteoclastogenesis in Girls and Young Women with Turner’s Syndrome. Bone.

[21] Zaidi M., Yuen T., Kim S.-M. (2023). Pituitary Crosstalk with Bone, Adipose Tissue and Brain. Nat. Rev. Endocrinol..

[22] Zaidi M., Kim S.-M., Ryu V., Lizneva D., Davies T.F., Rosen C.J., Yuen T., Giustina A. (2026). Neuroendocrine and Neural Control of Bone Mass in Health and Disease. J. Clin. Investig..

[23] Shi K., Liu L., He Y.-J., Li D., Yuan L.-X., Lash G.E., Li L. (2016). Body Composition and Bone Mineral Status in Patients with Turner Syndrome. Sci. Rep..

[24] Augoulea A., Zachou G., Lambrinoudaki I. (2019). Turner Syndrome and Osteoporosis. Maturitas.

[25] Gravholt C.H., Andersen N.H., Christin-Maitre S., Davis S.M., Duijnhouwer A., Gawlik A., Maciel-Guerra A.T., Gutmark-Little I., Fleischer K., Hong D. (2024). Clinical Practice Guidelines for the Care of Girls and Women with Turner Syndrome. Eur. J. Endocrinol..

[26] Klein K.O., Rosenfield R.L., Santen R.J., Gawlik A.M., Backeljauw P.F., Gravholt C.H., Sas T.C.J., Mauras N. (2018). Estrogen Replacement in Turner Syndrome: Literature Review and Practical Considerations. J. Clin. Endocrinol. Metab..

[27] Szybiak W., Kujawa B., Miedziaszczyk M., Lacka K. (2023). Effect of Growth Hormone and Estrogen Replacement Therapy on Bone Mineral Density in Women with Turner Syndrome: A Meta-Analysis and Systematic Review. Pharmaceuticals.

[28] Nishigaki S., Itonaga T., Hasegawa Y., Kawai M. (2021). Starting Age of Oestrogen-progestin Therapy Is Negatively Associated with Bone Mineral Density in Young Adults with Turner Syndrome Independent of Age and Body Mass Index. Clin. Endocrinol..

[29] Hasegawa Y., Ikegawa K., Munenaga T., Itonaga T., Mitani-Konno M., Kawai M., Amano N. (2025). Bone Health and Pubertal Induction in Turner Syndrome: The Possibility of Earlier Transdermal Lower-Dose Estradiol Therapy for Healthy Bone Density and Quality. Am. J. Med. Genet. C Semin. Med. Genet..

[30] Brander E.P.A., Keenahan L.A., Sangi-Haghpeykar H., Graham M., Dietrich J.E. (2023). The Effect of the Rate of Increase of Estrogen Replacement Therapy on Bone Mineral Density Accrual in Young Patients with Turner Syndrome. J. Pediatr. Adolesc. Gynecol..

[31] Ari M., Bakalov V.K., Hill S., Bondy C.A. (2006). The Effects of Growth Hormone Treatment on Bone Mineral Density and Body Composition in Girls with Turner Syndrome. J. Clin. Endocrinol. Metab..

[32] Backeljauw P., Blair J.C., Ferran J.-M., Kelepouris N., Miller B.S., Pietropoli A., Polak M., Sävendahl L., Verlinde F., Rohrer T.R. (2023). Early GH Treatment Is Effective and Well Tolerated in Children With Turner Syndrome: NordiNet^®^ IOS and Answer Program. J. Clin. Endocrinol. Metab..

[33] Zitzmann M., Aksglaede L., Corona G., Isidori A.M., Juul A., T’Sjoen G., Kliesch S., D’Hauwers K., Toppari J., Słowikowska-Hilczer J. (2021). European Academy of Andrology Guidelines on Klinefelter Syndrome Endorsing Organization: European Society of Endocrinology. Andrology.

[34] Mameli C., Street M.E., Vernice M., Tarani L., Valsecchi C., Fiore G., Lugarà C., Paparella R., Shulhai A.M., Pedretti P. (2026). Growing up with Klinefelter Syndrome: Challenges of Care in Children and Adolescents and Patients Advocacy. Endocrine.

[35] Stagi S., Di Tommaso M., Manoni C., Scalini P., Chiarelli F., Verrotti A., Lapi E., Giglio S., Dosa L., De Martino M. (2016). Bone Mineral Status in Children and Adolescents with Klinefelter Syndrome. Int. J. Endocrinol..

[36] Aksglaede L., Molgaard C., Skakkebaek N.E., Juul A. (2008). Normal Bone Mineral Content but Unfavourable Muscle/Fat Ratio in Klinefelter Syndrome. Arch. Dis. Child..

[37] Vogiatzi M.G., Davis S.M., Ross J.L. (2021). Cortical Bone Mass Is Low in Boys with Klinefelter Syndrome and Improves with Oxandrolone. J. Endocr. Soc..

[38] López Krabbe H.V., Holm Petersen J., Asserhøj L.L., Johannsen T.H., Christiansen P., Jensen R.B., Cleemann L.H., Hagen C.P., Priskorn L., Jørgensen N. (2023). Reproductive Hormones, Bone Mineral Content, Body Composition, and Testosterone Therapy in Boys and Adolescents with Klinefelter Syndrome. Endocr. Connect..

[39] Piot A., Plotton I., Boutroy S., Bacchetta J., Ailloud S., Lejeune H., Chapurlat R.D., Szulc P., Confavreux C.B. (2022). Klinefelter Bone Microarchitecture Evolution with Testosterone Replacement Therapy. Calcif. Tissue Int..

[40] Paparella R., Panvino F., Tarani F., Maglione V., Niceta M., Pucarelli I., Spaziani M., Tenuta M., Carlomagno F., Pozza C. (2026). Early Endocrine and Metabolic Profiles in Prepubertal Boys with 47,XXY Klinefelter Syndrome: A Retrospective Cross-Sectional Study. Ann. Endocrinol..

[41] Ferlin A., Selice R., Di Mambro A., Ghezzi M., Di Nisio A., Caretta N., Foresta C. (2015). Role of Vitamin D Levels and Vitamin D Supplementation on Bone Mineral Density in Klinefelter Syndrome. Osteoporos. Int. J. Establ. Result Coop. Eur. Found. Osteoporos. Natl. Osteoporos. Found. USA.

[42] Spiekermann J., Höppner J., Ibnukhsein E., Sinningen K., Hanusch B., Kiewert C., Siggelkow H., Grasemann C. (2024). Description of Bone Health in Adolescents and Young Persons with Klinefelter Syndrome—Results from a Pilot Study. Mol. Cell. Pediatr..

[43] Bojesen A., Birkebæk N., Kristensen K., Heickendorff L., Mosekilde L., Christiansen J.S., Gravholt C.H. (2011). Bone Mineral Density in Klinefelter Syndrome Is Reduced and Primarily Determined by Muscle Strength and Resorptive Markers, but Not Directly by Testosterone. Osteoporos. Int..

[44] Mehmet B., Dwyer A.A., Jayasena C.N., Gillard S., Llahana S. (2025). Update on Physical, Psychological, and Quality of Life Management in Klinefelter Syndrome. J. Clin. Endocrinol. Metab..

[45] Pizzocaro A., Vena W., Condorelli R., Radicioni A., Rastrelli G., Pasquali D., Selice R., Ferlin A., Foresta C., Jannini E.A. (2020). Testosterone Treatment in Male Patients with Klinefelter Syndrome: A Systematic Review and Meta-Analysis. J. Endocrinol. Investig..

[46] Pozza C., Sesti F., Tenuta M., Spaziani M., Tarantino C., Carlomagno F., Minnetti M., Pofi R., Paparella R., Lenzi A. (2023). Testicular Dysfunction in 47,XXY Boys: When It All Begins. A Semilongitudinal Study. J. Clin. Endocrinol. Metab..

[47] Otter M., Schrander-Stumpel C.T., Curfs L.M. (2010). Triple X Syndrome: A Review of the Literature. Eur. J. Hum. Genet..

[48] Tartaglia N.R., Howell S., Sutherland A., Wilson R., Wilson L. (2010). A Review of Trisomy X (47,XXX). Orphanet J. Rare Dis..

[49] Astro V., Cardona-Londoño K.Y., Cortés-Medina L.V., Alghamdi R., Ramírez-Calderón G., Kefalas F., Dilmé-Capó J., Radío S., Adamo A. (2025). An iPSC-Based Model of 47,XYY Jacobs Syndrome Reveals a DNA Methylation-Independent Transcriptional Dysregulation Shared with Male X Aneuploid Cells. Genome Res..

[50] International Society for Clinical Densitometry (2019). 2019 ISCD Official Positions: Pediatric Skeletal Health Assessment in Children from Infancy to Adolescence.

[51] Bachrach L.K., Gordon C.M. (2016). Section on Endocrinology. Bone Densitometry in Children and Adolescents. Pediatrics.

[52] Ciancia S., Van Rijn R.R., Högler W., Appelman-Dijkstra N.M., Boot A.M., Sas T.C.J., Renes J.S. (2022). Osteoporosis in Children and Adolescents: When to Suspect and How to Diagnose It. Eur. J. Pediatr..

[53] American College of Radiology, Society for Pediatric Radiology, Society of Skeletal Radiology (2023). ACR–SPR–SSR Practice Parameter for the Performance of Quantitative Computed Tomography (QCT) Bone Mineral Density.

[54] Rubin K. (1998). Turner Syndrome and Osteoporosis: Mechanisms and Prognosis. Pediatrics.

[55] Golden N.H., Abrams S.A., Committee on Nutrition (2014). Optimizing Bone Health in Children and Adolescents. Pediatrics.

[56] Paparella R., Panvino F., Rasio D., Tarani F., Niceta M., Leonardi L., Pucarelli I., Pancheva R., Fiore M., Tarani L. (2026). Clinical Features of Hypo- and Hypervitaminosis of Fat-Soluble Vitamins in Pediatric Patients. Curr. Med. Chem..

[57] Rizzoli R., Bianchi M.L., Garabédian M., McKay H.A., Moreno L.A. (2010). Maximizing Bone Mineral Mass Gain during Growth for the Prevention of Fractures in the Adolescents and the Elderly. Bone.

[58] Baroncelli G.I., Tessaris D., Aversa T., Bertelli E., Comberiati P., Corica D., Di Iorgi N., Faienza M.F., Fintini D., Franceschi R. (2026). Practical Approach to the Diagnosis, Management, and Treatment of Pediatric Patients with Bone Fragility: An Expert Opinion. J. Endocrinol. Investig..

[59] Cannalire G., Biasucci G., Bertolini L., Patianna V., Petraroli M., Pilloni S., Esposito S., Street M.E. (2024). Osteoporosis and Bone Fragility in Children: Diagnostic and Treatment Strategies. J. Clin. Med..

